# Development of a national childhood obesogenic environment index in the United States: differences by region and rurality

**DOI:** 10.1186/s12966-020-00984-x

**Published:** 2020-07-02

**Authors:** Andrew T. Kaczynski, Jan M. Eberth, Ellen W. Stowe, Marilyn E. Wende, Angela D. Liese, Alexander C. McLain, Charity B. Breneman, Michele J. Josey

**Affiliations:** 1 Department of Health Promotion, Education and Behavior, Arnold School of Public Health, University of South Carolina, Columbia, SC 29208 USA; 2 Prevention Research Center, Arnold School of Public Health, University of South Carolina, Columbia, SC 29208 USA; 3 Department of Epidemiology and Biostatistics, Arnold School of Public Health, University of South Carolina, Columbia, SC 29208 USA; 4 Rural and Minority Health Research Center, Arnold School of Public Health, University of South Carolina, Columbia, SC 29208 USA

**Keywords:** Childhood obesity, Environment, Measurement, Physical activity, Healthy eating

## Abstract

**Background:**

Diverse environmental factors are associated with physical activity (PA) and healthy eating (HE) among youth. However, no study has created a comprehensive obesogenic environment index for children that can be applied at a large geographic scale. The purpose of this study was to describe the development of a childhood obesogenic environment index (COEI) at the county level across the United States.

**Methods:**

A comprehensive search of review articles (*n* = 20) and input from experts (*n* = 12) were used to identify community-level variables associated with youth PA, HE, or overweight/obesity for potential inclusion in the index. Based on strength of associations in the literature, expert ratings, expertise of team members, and data source availability, 10 key variables were identified – six related to HE (# per 1000 residents for grocery/superstores, farmers markets, fast food restaurants, full-service restaurants, and convenience stores; as well as percentage of births at baby (breastfeeding)-friendly facilities) and four related to PA (percentage of population living close to exercise opportunities, percentage of population < 1 mile from a school, a composite walkability index, and number of violent crimes per 1000 residents). Data for each variable for all counties in the U.S. (*n* = 3142) were collected from publicly available sources. For each variable, all counties were ranked and assigned percentiles ranging from 0 to 100. Positive environmental variables (e.g., grocery stores, exercise opportunities) were reverse scored such that higher values for all variables indicated a more obesogenic environment. Finally, for each county, a total obesogenic environment index score was generated by calculating the average percentile for all 10 variables.

**Results:**

The average COEI percentile ranged from 24.5–81.0 (M = 50.02,s.d. = 9.01) across US counties and was depicted spatially on a choropleth map. Obesogenic counties were more prevalent (F = 130.43,*p* < .0001) in the South region of the U.S. (M = 53.0,s.d. = 8.3) compared to the Northeast (M = 43.2,s.d. = 6.9), Midwest (M = 48.1,s.d. = 8.5), and West (M = 48.4,s.d. = 9.8). When examined by rurality, there were also significant differences (F = 175.86,*p* < .0001) between metropolitan (M = 46.5,s.d. = 8.4), micropolitan (M = 50.3,s.d. = 8.1), and rural counties (M = 52.9,s.d. = 8.8) across the U.S.

**Conclusion:**

The COEI can be applied to benchmark obesogenic environments and identify geographic disparities and intervention targets. Future research can examine associations with obesity and other health outcomes.

## Background

Childhood obesity has become a major threat to public health in the United States (US) and other developed countries [1, 2]. Within the last three decades, child obesity rates have more than tripled, such that approximately 17% of children aged 2 to 19 years are obese and 32% are overweight or obese [3]. The consequences of childhood obesity can be severe and long-lasting, as obese children have an increased risk for high blood pressure, high cholesterol, type 2 diabetes, asthma, sleep apnea, and fatty liver disease [4–9]. Further, obese children are more likely to become obese adults, [2, 9] and obese adults are at an increased risk for morbidity from hypertension, stroke, and some cancers [10, 11].

Although the physiological causes of excess weight gain are complex, obesity is generally the result of an energy imbalance, caused by energy intake (i.e., caloric consumption) exceeding energy expenditure (i.e., physical activity) [2, 12–15]. Much research has suggested that the environment is a key factor contributing to unhealthy diets and physical inactivity [16, 17]. For example, access to healthy foods, such as proximity to supermarkets, and availability of unhealthy foods, such as proximity to fast food restaurants and convenience stores, can influence diet and weight status [18, 19]. Further, access to recreation facilities, such as parks and playgrounds, other neighborhood factors such as walkability and safety, and ability to utilize active transportation to work or school are associated with increased physical activity (PA) [20, 21].

In recognition of the influence of environmental factors on obesity-related health behaviors, researchers have increasingly focused on what has been termed the “obesogenic environment” [17, 22, 23]. Swinburn et al. originally defined an obesogenic environment as “the sum of influences that the surroundings, opportunities, or conditions of life have on promoting obesity in individuals or populations” (p. 564) [24]. Similarly, Gauthier and Krajicek developed a definition of an obesogenic environment for children: “instances where a child is placed into a situation, circumstance, or surrounding where there exists the opportunity to choose, engage in, or be influenced by internal (i.e., within the home) or external structures (i.e., outside the home) where the aggregate effects promote (or result in) an abnormal, or elevated, BMI percentile” (p. 205) [25]. In spite of these useful broader conceptualizations, others have more narrowly operationalized the obesogenic environment through community elements that make up the built PA and nutrition environments [22]. For example, Frank et al. described the development of a GIS-based multicomponent child obesogenic environment measure for San Diego and Seattle regions comprised of select elements related to PA (walkability, parks) and nutrition (presence and density of fast food restaurants and distance to supermarkets) [26]. Other researchers, while not proposing obesogenic environment indices, have nevertheless investigated diverse environmental influences on childhood obesity at varying scales within communities, including factors such as neighborhood safety, access to and quality of parks and green spaces, housing density, land use mix, access to destinations, traffic, transit opportunities, and recreation facilities, as well as supermarkets, farmers markets, and fast food outlets [26–30].

For the purposes of this study, obesogenic environments are defined as the sum of physical elements within communities that promote sedentarism, restrict PA, and encourage unhealthy eating practices among children. Despite substantial research into environmental influences on childhood obesity, no prior studies have sought to develop a comprehensive community obesogenic environment index for children that can be applied across a large geographic scale. Creating such an index would allow for comparisons across counties and regions and would help identify index components of greatest concern and action areas to prioritize. It would also facilitate research in which the obesogenic environment index is examined as a comparative or additive measure to more micro-level influences, such as personal factors or the home environment. Moreover, the development of an obesogenic environment index would facilitate identification of high-risk locations and improve allocation of resources to address environmental justice disparities in access to healthy food, PA-promoting amenities, or both. Given these considerations, the purposes of this study were to 1) describe the development of a childhood obesogenic environment index (COEI), and 2) examine differences in obesogenic environment index values by region and rurality across the US.

## Methods

### Development of childhood obesogenic environment index

Development of the childhood obesogenic environment index (COEI) was accomplished using an a priori approach, which integrates theory and existing, empirical knowledge [31, 32]. In particular, we adopted a social ecological approach to health promotion, which places particular emphasis on environmental influences on PA, healthy eating (HE), and obesity [33], paired with published literature on obesogenic environments for children and the expertise of our research team. Specifically, index development consisted of a series of stages involving 1) a review of extant literature, 2) expert feedback, and 3) data sourcing and analyses.

Initially, to identify potential elements to include within a preliminary version of the index, a search was conducted to locate review articles on environmental factors related to youth PA and nutrition. The search was limited to review articles published between January 1999 (when obesogenic environments were first coined) and December 2017 and was conducted on PubMed. Search terms were modelled after those used in other similar reviews [34] and included 11 terms related to obesity or overweight status (e.g., obesity, adiposity, body mass index), 69 terms related to the built environment (e.g., environmental influence, neighborhood characteristics, food outlet, walkability), and 6 terms related to the target population of young people (e.g., adolescence, youth, childhood). The search returned 3983 articles, which were narrowed to 49 after a detailed review of each manuscript’s title and abstract. In a final assessment that involved reading each article in full, 20 articles were found to meet the inclusion criteria of being a review article that focused on one or more elements of community nutrition or PA environments and contained some or all results describing relationships with youth PA, HE, and/or weight status [20, 27, 35–52].

This review, combined with the knowledge and experience of project team members, identified approximately 100 unique PA and nutrition environmental variables. To aid in organizing and narrowing down the list, similar variables were sorted into categories (e.g., school access, parks and recreation facilities, walkability/transport, food resource access). For example, related variables such as park access, proximity to playgrounds, availability of green space, distance to recreation facilities were all grouped to identify the construct(s) important for inclusion in the index. Several criteria were then applied in evaluating the variable for further review, including whether it related to access to a community physical environment structure, if it was an objective measure (rather than a latent construct or based on perceptions), the strength of its association with childhood PA, HE, or obesity, and whether the variable could likely be collected at the census tract or county level. We did not restrict variables of interest to child-specific measures, and many of the variables may also be important environmental obesity determinants for adults.

After extensive discussion among the study team, a final list of 24 variables was agreed upon for distribution to additional experts within the fields of PA, nutrition, and environmental influences on obesity. Twelve of 16 invited experts agreed to participate and were asked to rate the importance of each of the 24 variables (1 = low importance, 7 = high importance), to provide input on potential data sources that had been identified for each variable, and to offer any additional comments about the variables, data sources, or other aspects of the index development process. Experts were also able to suggest additional variables and data sources not included within the original list.

Expert feedback on the 29 variables (including 5 suggested by the experts) was evaluated by the project team, and consensus was obtained regarding variables to include in the index. Specifically, mean ratings and standard deviations were calculated for each variable and used as one indicator of variable importance. Likewise, expert reviewers provided valuable comments for evaluating factors such as the reliability or validity of a variable or its potential data source and the availability of a variable at a specific geographic level nationwide. Ultimately, extensive discussion of variable ratings, expert reviewer feedback, and data source availability among the project team resulted in a refined list of 10 variables to be included in the COEI.

Table 1 provides definitions and data source descriptions for the 10 obesogenic environment index variables. Briefly, the number of fast food restaurants (limited-service restaurants where patrons typically pay prior to receiving food; e.g., McDonald’s), full-service restaurants (full-service restaurants where patrons are served seated and pay after receiving food; e.g., Chili’s), convenience stores (outlets selling a limited number of food items but include milk, bread, soda, and snacks; e.g., Quick-Trip) grocery stores (outlets selling general food items including fresh and frozen foods, fruits and vegetables, and prepared meats; e.g., Kroger)/superstores (outlets selling general food items in addition to other non-food household items; e.g., CostCo), and farmers markets (outlets where two or more vendors sell agricultural products directly to consumers) per 1000 residents for each county were collected from the United States Department of Agriculture (USDA) [53]. An additional nutrition variable important for childhood obesity is the percentage of births occurring at baby-friendly hospitals, a measure of breastfeeding support [54, 55]. This was collected at the state level (due to unavailability at the county level) from the Centers for Disease Control and Prevention [56].

**Table 1:** Variables Included in Childhood Obesogenic Environment Index

VARIABLE	MEASURE	SOURCE	YEAR
**FAST FOOD RESTAURANTS**	Number of fast food restaurants (NAICS 722211) in the county per 1000 county residents	United States Department of Agriculture^1^ (Restaurants: U.S. Census Bureau, County Business Patterns; Population: U.S. Census Bureau, Population Estimates)	2014
**FULL-SERVICE RESTAURANTS**	Number of full-service restaurants (NAICS 7221110) in the county per 1000 county residents	United States Department of Agriculture^1^ (Restaurants: U.S. Census Bureau, County Business Patterns; Population: U.S. Census Bureau, Population Estimates)	2014
**GROCERY STORES AND SUPERCENTERS**	Number of grocery stores/supermarkets and supercenters/warehouse club stores (NAICS 445110 & 452,910) in the county per 1000 county residents	United States Department of Agriculture^1^ (Stores: U.S. Census Bureau, County Business Patterns; Population: U.S. Census Bureau, Population Estimates)	2014
**FARMERS MARKETS**	Number of farmers markets (NAICS 115114) in the county per 1000 county residents	United States Department of Agriculture^1^ (Farmers Markets: Agricultural Marketing Service, Marketing Services Division Population: U.S. Census Bureau, Population Estimates)	2016
**CONVENIENCE STORES**	Number of convenience stores (NAICS 445120 & 447,110) in the county per 1000 county residents	United States Department of Agriculture^1^ (Stores: U.S. Census Bureau, County Business Patterns Population: U.S. Census Bureau, Population Estimates)	2014
**BIRTHS AT BABY-FRIENDLY FACILITIES**	Percent births at baby-friendly facilities at the state level	Centers for Disease Control and Prevention^2^ (Breastfeeding Report Card, Division of Nutrition, Physical Activity, and Obesity, National Center for Chronic Disease Prevention and Health Promotion)	2016
**EXERCISE OPPORTUNITIES**	Percentage of those with access, defined as residing in a census block within a half mile of a park (NAICS 712190)**,** residing in an urban census block within one mile of a recreational facility (NAICS 713940), or residing in a rural census block within three miles of a recreational facility	County Health Rankings^3^ (2010 US Census Bureau Population data, 2016 SIC codes, 2016 parks, Business Analyst, Delorme map data, ESRI, US Census TIGER/Line Files)	2018
**VIOLENT CRIME**	Number of violent crimes reported per 100,000 population	County Health Rankings^4^ (Uniform Crime Reporting, Federal Bureau of Investigation)	2012–2014
**WALKABILITY**	National Walkability Index	EPA Smart Growth Smart Location Mapping Database^5^	2010–2012
**SCHOOL PROXIMITY**	Percentage of the county covered by ½ mile school buffers. A half-mile buffer was created around each public school location and then the square mileage covered by the school buffers was aggregated to the county level. Total area covered by these school buffers was divided by total area of the county to obtain the percentage of the county that was within close proximity to a school.	National Center for Education Statistics^6^	2016–2017

1. U.S. Department of Agriculture, Food Environment Atlas: [53] https://www.ers.usda.gov/data-products/food-environment-atlas/documentation/

2. Centers for Disease Control and Prevention, Breastfeeding Report Card: [56] https://www.cdc.gov/breastfeeding/pdf/2016breastfeedingreportcard.pdf

3. County Health Rankings, Access To Exercise Opportunities: [57] https://www.countyhealthrankings.org/explore-health-rankings/measures-data-sources/county-health-rankings-model/health-factors/health-behaviors/diet-exercise/access-to-exercise-opportunities

4. County Health Rankings, Violent Crime Rate: [58] https://www.countyhealthrankings.org/explore-health-rankings/measures-data-sources/county-health-rankings-model/health-factors/social-and-economic-factors/community-safety/violent-crime-rate

5. U.S. Environmental Protection Agency, Smart Location Mapping, National Walkability Index: [59] https://www.epa.gov/smartgrowth/smart-location-mapping#walkability

6. National Center For Education Statistics, Education Demographic And Geographic Estimates, School Locations And Geoassignments: [60] https://nces.ed.gov/programs/edge/geographic/schoollocations

Physical activity variables within the index are also shown in Table 1. Data on the percent of individuals with access to exercise opportunities were ascertained from County Health Rankings. Access to exercise opportunities (e.g., parks, recreation facilities) constituted residing within a census block within a half mile of a park, residing in a urban census block within one mile of a recreation facility, or residing in a rural census block that is within three miles of a recreational facility [57]. Information on population-weighted violent crime incidence (e.g., homicide, robbery) was also collected from County Health Rankings (originally obtained from Uniform Crime Reporting Program Data) as a marker of safety [58]. County-level population-weighted walkability ratings were represented using the National Walkability Index based on the Environmental Protection Agency’s Smart Location Mapping Database, aggregated from the original census block group level [59]. This measure takes into consideration multiple aspects of walkability, including street intersection density, predicted commute mode, and employment types. Finally, a proximity to schools variable was created using location data for all public schools from the National Center for Education Statistics [60]. Specifically, using ArcGIS Pro, the percentage of each county’s area that was within at least one half-mile of a school was calculated by the project team.

For each variable, the values for all counties in the US (*N* = 3142) were ranked and a percentile was assigned to each county that ranged from 0 to 100 (0 = least obesogenic, 100 = most/worst obesogenic). Variables that were considered positive aspects of the environment – grocery stores/superstores, farmers markets, births at baby-friendly hospitals, exercise opportunities, school proximity, and walkability – were reverse scored such that a lower score for these variables indicated a healthy environment. Variables that were considered negative aspects of the environment – fast food restaurants, full-service restaurants, convenience stores, and violent crime – were scored as is, such that a higher score for these variables indicated an unhealthy environment. For each county, a total COEI score was generated by calculating the average percentile for all 10 variables, with higher COEI scores indicating more obesogenic (worse) environments. Minimal missing data were excluded such that if a variable(s) was not available for a county, the total score was generated taking the mean of all available variables.

#### Study setting

Each county in the US was classified by region and rurality. U.S. Census regions were used to classify counties into four groups: Northeast, Midwest, South, and West [61]. Urban Influence Codes (UIC) were collected from the USDA and the original 12 categories were aggregated to classify counties as either metropolitan, micropolitan, or rural [62–64]. Metropolitan counties were conceptualized as those in large metropolitan areas of 1+ million residents or as those in small metropolitan areas of less than 1 million residents (UIC codes 1, 2). Micropolitan counties were those in micropolitan areas and included those adjacent to either a large metropolitan area or a small metropolitan area (UIC codes 3, 5, & 8). Finally, rural counties were those considered non-core, including those adjacent to large or small metropolitan or micropolitan areas (UIC codes 4, 6, 7 & 9–12).

#### Analyses

Descriptive statistics and choropleth maps were used to characterize the COEI for all counties across the US. Analysis of variance was used to compare COEI scores by region and rurality. All analyses were conducted in ArcMap™ (ESRI, Redlands CA) and SAS 9.4 (Cary, NC). Tests were considered significant at *p* < .05.

## Results

**Fig. 1:**
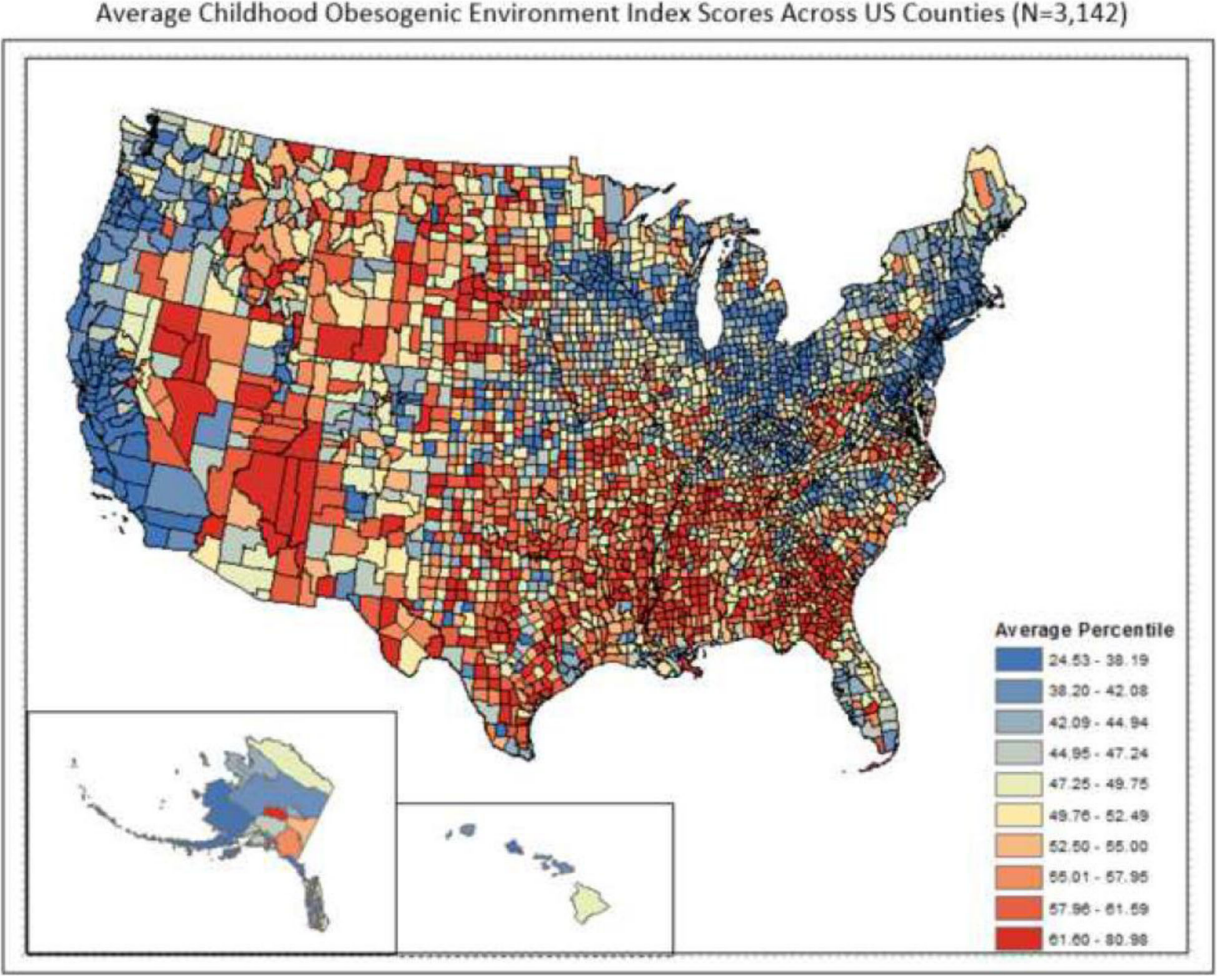
Average Childhood Obesogenic Environment Index Scores Across US Counties

Across all counties in the US (*N* = 3142), the average COEI percentile ranged from 24.53–80.98 (Mean [M] = 50.02, Standard Deviation [SD] = 9.01), with lower scores indicating a less obesogenic environment and higher scores indicating a more obesogenic environment. Figure 1 displays the COEI scores by county across the US. Visually, there were fewer obesogenic counties along coastal areas of the north and west, and in areas around the Great Lakes. More obesogenic (or less healthy) counties were in central areas of the south and midwest, and proximal to the Rocky Mountains.

When comparing COEI scores by formal US regions (Table 2), there were significant differences between the Northeast, Midwest, South, and West regions (F = 130.43, *p* < .0001). The Northeast region (M = 43.2, SD = 6.9) had a significantly lower (better) average index value compared to Midwest (M = 48.1, SD = 8.5), South (M = 53.0, SD = 8.3), and West (M = 48.4, SD = 9.8) regions. The Midwest region showed a significantly higher (worse) average index score compared to the Northeast region and a significantly lower (better) index score compared to the South region, but no significant difference compared to the West region. Counties in the South region had significantly higher (worse) index scores compared to all other regions.

**Table 2:** Childhood Obesogenic Environment Index by Region and Rurality

	AVERAGE PERCENTILE (SD)	MEDIAN PERCENTILE	RANGE
**NORTHEAST**	43.2^a^ (6.9)	43.1	25.4–63.0
**MIDWEST**	48.1^b^ (8.5)	47.5	26.4–79.9
**SOUTH**	53.0^c^ (8.3)	53.4	28.9–77.8
**WEST**	48.4^b^ (9.8)	47.7	24.5–81.0
**ANOVA (F)**	130.43		
**ANOVA (P)**	<.0001		
**METROPOLITAN COUNTIES**	46.5^a^ (8.4)	46.1	25.4–81.0
**MICROPOLITAN COUNTIES**	50.3^b^ (8.1)	49.9	29.9–73.3
**RURAL COUNTIES**	52.9^c^ (8.8)	52.9	24.5–79.9
**ANOVA (F)**	175.86		
**ANOVA (P)**	<.0001		

Notes

A larger percentile indicates a more obesogenic environment

^a,b,c^Different superscript letters indicate means that were significantly different at *p* < .05

When examined by county rurality (Table 2), there were significant differences between metropolitan, micropolitan, and rural counties across the US (F = 175.86, *p* < .0001). Specifically, metropolitan counties had significantly lower (better) obesogenic environment index scores (M = 46.5, SD = 8.4), compared to micropolitan (M = 50.3, SD = 8.1) and rural (M = 52.9, SD = 8.8) counties. Similarly, micropolitan areas had significantly lower (better) index scores compared to rural areas (Table 2).

Finally, analyses were also conducted incorporating both county region and rurality (Table 3) and this interaction was significant (F = 82.07, *p* < .0001). Overall, the Northeast region had the lowest (best) childhood obesogenic environment index for all of metropolitan (M = 40.5), micropolitan (M = 45.1), and rural (M = 49.4) counties. In contrast, the South had the highest (worst) index values across metropolitan (M = 50.1), micropolitan (M = 54.8), and rural (M = 55.2) counties. Looking at rurality differences within each region, important differences were observed. There were significant differences between all categories of rurality in the Northeast (F = 38.31, *p* < .0001), Midwest (F = 89.81, *p* < .0001), and West (F = 40.19, *p* < .0001), with a consistent order of metropolitan (best) followed by micropolitan and then rural counties. In the South, there were also overall significant differences based on rurality (F = 67.88, *p* = <.0001): metropolitan counties had better environments (M = 50.1), while micropolitan (M = 54.8) and rural (M = 55.2) areas were not significantly different.

**Table 3:** Childhood Obesogenic Environment Index by County Rurality and Region

	NORTHEAST MEAN (SD)	MIDWEST MEAN (SD)	SOUTH MEAN (SD)	WEST MEAN (SD)
**METROPOLITAN COUNTIES**	40.5^a^ (6.3)	43.7^a^ (7.3)	50.1^a^ (7.6)	43.0^a^ (8.9)
**MICROPOLITAN COUNTIES**	45.1^b^ (5.4)	47.0^b^ (7.4)	54.8^b^ (7.0)	48.9^b^ (7.8)
**RURAL COUNTIES**	49.4^c^ (5.0)	51.1^c^ (8.3)	55.2^b^ (8.7)	51.8^c^ (9.6)
**ANOVA (F)**	38.31	89.81	67.88	40.19
**ANOVA (P)**	<.0001	<.0001	<.0001	<.0001
**ANOVA INTERACTION TERM (F)**	82.07			
**ANOVA INTERACTION TERM (P)**	<.0001			

Notes

A larger percentile indicates a more obesogenic environment

^a,b,c^Different superscript letters indicate means that were significantly different at *p* < .05

## Discussion

This study described the first known attempt at developing a comprehensive yet parsimonious obesogenic environment index for the US oriented toward youth. Such efforts are critical as the personal and societal costs of obesity continue to grow and as environments are increasingly recognized as both contributing to and as potential solutions to this medical and financial crisis [17]. Some past research has accomplished a similar process at more local levels (e.g., one or two cities) using a more limited set of variables [26, 65]. For example, to describe youth obesogenic environments, indicators of the physical activity and nutrition environments were compiled for two major-metropolitan cities (San Diego and Seattle areas) [26]. This work differed from the present study as it utilized local data on multiple components specific to walkability and to proximity to healthy food. Similarly, additional research has also incorporated local-level data to examine childhood obesogenic environments in a Southeastern county utilizing detailed information on physical activity (e.g., park quality) and nutrition (e.g., fast-food restaurants near the home) and assessed this data in relation to childhood obesity [65]. However, documenting the status of obesogenic environments at the national scale can highlight widespread disparities in access to PA and/or HE resources and potentially lead to physical and policy changes to address such inequities.

Two main approaches to index development are commonly used – those that employ an a priori approach (i.e., based on theory and literature) and those that use an a posteriori approach (i.e., informed by statistical analyses) [31, 32, 66]. One key advantage of the a priori approach is that it focuses on a comprehensive list of elements that are established in past literature, and it has been paramount for development of other composite obesity-related metrics, such as the *Healthy Eating Index* (HEI) [67, 68]. For example, the HEI includes relevant healthy and unhealthy food outlets to conceptualize “access to healthy food resources” and “exposure to unhealthy, calorie-dense food options” rather than considering only a selective list of variables that show strong correlations with one another. In contrast, indices such as those developed to characterize area-based (e.g., neighborhood) socioeconomic disadvantage often include multiple indicators (e.g., education, income, employment, home ownership) that are highly related and would be expected to co-vary [69–71]. The COEI described here is more similar to something like the County Health Rankings that integrate diverse, key metrics derived from theory and literature (e.g., low birthweight, physical inactivity, flu vaccinations, violent crime, commuting distance) into an overall measure of community health and are widely adopted in the U.S. [72, 73]. Unlike an a posteriori approach, our a priori approach builds on previous research and does not disregard the extensive published literature and current paradigms for defining obesogenic environments.

Ultimately, the COEI combined ten variables deemed essential to assessing environmental supports (or lack thereof) for PA and HE according to a review of existing literature, ratings and feedback by expert reviewers, and the extensive knowledge and experience of our diverse study team. When aggregated into a composite score for each county, index values showed substantial variability across the US (ranging from 24 to 81 out of 100) and somewhat distinct patterns of geographic clustering [74]. For example, the vast majority of counties in the South region displayed COEI scores in the worst three deciles. This was confirmed by analyses that found that South region counties had significantly higher obesogenic environment values compared to the other three regions. This is not dissimilar to other research, which has documented higher rates of adult obesity in Southern areas [63] and related predictors such as lesser access to recreational facilities, rurality, and residential segregation [75]. In contrast, the Northeast region had COEI scores almost ten points better than the South, and the western coast of the US was also among the top two deciles of counties for advantageous environments. Other research focusing on either a limited number of locations (e.g., cities or states) or individual variables (e.g., parks, fast food restaurants) has likewise documented resource disparities with respect to PA and HE environments [76–80]. More regional and national studies are needed that ascertain the historical, cultural, political, planning, and economic factors that have contributed to such differences. These mechanisms, which scale across local, state, and national levels, may be leveraged going forward to reduce widespread disparity across regions and the entire nation.

Significant differences by county rurality across the US were also revealed. Overall, counties classified as metropolitan (areas with 1+ million residents or small metropolitan areas) had significantly lower (better) COEI scores compared to micropolitan and rural counties, which were more similar (but with micropolitan still significantly better than rural). This is consistent with conclusions from past studies that rural locations have reduced access to PA and HE environmental supports [39, 81]. Rural areas experience greater concerns than more urban areas with respect to lower rates of PA or HE and with problematic health outcomes such as obesity and related chronic diseases [82–84]. This study highlights, on a national scale, that efforts to improve rural and micropolitan environments for PA and HE are needed. Such efforts might include infrastructure adaptations such as sidewalks and parks, policy strategies such as zoning of fast food restaurants or incentives to grocery stores, or programming and partnerships such as Safe Routes to School and farm to school initiatives [85–89]. Finally, analyses according to both region and rurality revealed that rural counties had more obesogenic environments across all regions. Moreover, rural and micropolitan counties of the South had the highest COEI scores compared to any other region and rurality distinctions.

### Limitations

This study had several important limitations. First, because most relevant data are not available at a more finite scale, the COEI was developed at the county level and it is possible that a smaller geographic area (e.g., census tract) better represents the local sphere of influence on obesogenic behaviors. However, developing the index at the county level is valuable to identify contextual factors and foster important environmental and policy intervention tactics that often occur at larger scales than neighborhoods or census tracts/block groups (e.g., school siting, food outlet zoning). Moreover, county-level attributes impact and are related to those at smaller units, and more rural counties may not even have smaller administrative units that influence PA and food environment decision-making. Further, influences on PA and HE may extend well outside an individual’s neighborhood into the broader county; for example, residents often travel several miles beyond their closest supermarket to shop for food [90–92]. Second, for many similar reasons, we limited the inclusion of key variables to those for which county-level data were available; other environmental information (e.g., quality of PA and food resources) may be valuable to merge into the index as data about obesogenic environments become better documented. Third, some debate continues about the importance of environmental influences, or specific variables, on obesity-related behaviors and outcomes. For example, some studies have failed to observe impacts of improving food environments on dietary intake or obesity [93, 94], but have shown that there are likely indirect effects of the built food environment on fruit and vegetable consumption via constructs such as perceptions (e.g., awareness) and actions such as food shopping behaviors (e.g., frequency) [93, 95, 96]. Fourth, all variables within the COEI were weighted equally. This decision was made in the absence of data or rationale to aggregate them otherwise, but future researchers and policymakers may wish to give more importance to certain factors in specific locations. Fifth, the COEI was not validated against a health behavior or outcome, such as childhood obesity prevalence. At present, such data are not reliably available at the county level across the US, but this represents an important step for future research when such small area estimates become available. Nonetheless, it is noteworthy that our index reasonably mirrored geographic patterns of adult obesity, with poorer environments documented in the South region and better environments found in the Northeast region and along the western coast [63]. Moreover, the variables contained in the index have been related to childhood obesity on more local levels so demonstrating how widespread these obesogenic features are across all U.S. counties may be valuable for public health intervention and surveillance. Sixth, our analyses were conducted using four US regions and three categories of rurality, but other useful ways of grouping counties may exist (e.g., nine census divisions). Finally, although the data utilized in this study are publicly available in the U.S., the index variables may not be accessible in other settings (e.g., census tract level) or in other countries, making more detailed or cross-country comparisons unachievable.

Further, there are limitations inherent to many of the individual index variables. Fast food restaurants, full-service restaurants, and convenience stores were considered negative aspects of the environment, but some healthful foods are available at these food outlets; likewise, grocery stores have a greater proportion of healthy options, but also many calorically dense products as well. Also, the percentage of births at baby-friendly facilities variable was gathered at the state rather than county level, which precludes any county variability. Violent crime may fail to capture perceived crime or non-violent crime that could influence PA. Finally, school proximity utilized public elementary and high-schools and excluded private school. Despite these limitations, there are major strengths associated with the index variables, in that all variable data are free and publicly available and most data can be gathered at multiple time points for longitudinal analyses.

## Conclusions

This study developed a novel childhood obesogenic environment index and highlighted important regional and rurality differences across US counties. However, several important opportunities exist for future research using these and similar data. For example, comparing the COEI against relevant outcomes will be valuable once stable estimates of childhood obesity data are available at the county level nationwide. In the meantime, other analyses could examine disparities in COEI values based on other known correlates of obesity such as county-level income, racial/ethnic composition, or residential segregation. In addition, undertaking a similar process using data about the existence of policies in states and communities (e.g., menu labeling, SNAP/EBT vouchers at stores and markets, joint/shared use of schools, complete streets) could lead to a policy environment index that may help explain the environmental disparities reported in our study. Future studies should explore the unique and possibly synergistic contributions of the obesogenic environment and other known predictors (e.g., family socioeconomic status) on childhood obesity at the individual level. Challenges to such an analysis include a lack of publicly available survey data with linked geographic identifiers, sampling constraints that ensure robust geographic heterogeneity in survey participants, and a need to address the uncertain geographic context problem through examining varying area-level effects [97]. Lastly, this study highlights the growing need for public health intervention in the Southern region of the US, particularly in non-metropolitan counties. Many behavioral interventions have targeted cultural norms or lack of education about healthy living in this region but disregard important drivers such as environmental supports for healthy behaviors or residential segregation that create greater access disparities [98]. Overall, future research and practice must continue to monitor and address the distribution of obesogenic environments in order to target childhood obesity disparities nationwide.

## Data Availability

The datasets used and/or analysed during the current study are available from the corresponding author on reasonable request. All sources for data generated or analysed during this study are included in this published article in Table 1. Additional methods for creating variables for analysis are available in the methods text.

## Abbreviations

US: United States
PA: Physical activity
UIC: Urban influence codes
USDA: United States department of agriculture
